# Longitudinal relationships between youth assets, seat belt use as a rider, and the environment

**DOI:** 10.1093/eurpub/ckac131.355

**Published:** 2022-10-25

**Authors:** E Tolma, S Vesely, R Oman, L Boeckman, C Aspy

**Affiliations:** 1 Education Sciences, European University Cyprus, Nicosia, Cyprus; 2 School of Public Health, University of Oklahoma Health Sciences Center, Oklahoma City, USA; 3 School of Public Health, University of Nevada, Reno, USA; 4 School of Medicine, University of Oklahoma Health Sciences Center, Oklahoma City, USA

## Abstract

**Background:**

Positive youth development theory can provide an alternative approach to promote Seat Belt Use while Riding in a Car (SBURC) among youth. The study aims to explore the relationship between youth assets, and the neighborhood environment in predicting SBURC.

**Methods:**

The Youth Asset Study (YAS) is a 4-year (5 waves) longitudinal study of a random sample of 1,111 youth (12-17 years old) and their parents, taking place in a Midwestern city, USA from 2003-2008. Seventeen youth assets were developed. The environment was measured objectively via the broken windows survey and subjectively via parents’ interviews. Assets and environmental factors at Waves 1-4 predicted SBURC at Waves 4-5 while controlling for demographics. Data were analyzed via marginal logistic regression and generalized estimated equations analyses.

**Results:**

The sample consisted of 1001 youth: 53% female; mean age=14.36 (SD = 1.59); 50% with income <$35,000; and 69% two-parent families. The proportion of youth not wearing a seat belt while riding in a car decreased from wave 4 to 5 among all youth. Individually, most assets had a positive relationship with SBURC (ORs ranged from 1.4 to 2.0), with parental monitoring, responsible choices, general aspirations for the future and good health practices having the largest ORs [2.0, 95% CI (1.5, 2.7); 1.9, 95% CI (1.5, 2.6); 1.8, 95% CI (1.2,2.8); 1.8, 95% CI (1.5,2.3)] respectively. Youth with a higher (≥ 12) number of assets were almost twice as likely to use a SBURC than those with a lower (^<^12) number [OR = 1.9, 95% CI (1.5, 2.3)]. Only one environmental factor, had a significant effect on SBURC [(OR = 0.64, 95% CI (0.45, 0.90)]. Assets retained their impact on SBURC, controlling for the environmental and demographic factors.

**Conclusions:**

Youth assets can be an alternative approach to promote SBURC among teens with emphasis on promoting parental monitoring, responsible choices, general aspirations for the future, and good health practices.

**Key messages:**

• Building youth assets can be another mechanism to promote seat belt use while driving among youth.

• The neighborhood environment might not be as important as other factors are in the promotion of seat belt use while driving.

